# Automated prediction of dosimetric eligibility for hypofractionated prostate radiotherapy

**DOI:** 10.1002/acm2.12198

**Published:** 2017-10-04

**Authors:** Anthony Lausch, Michael Lamey, Grace G. Zeng

**Affiliations:** ^1^ Department of Radiation Oncology University of Toronto Toronto ON Canada; ^2^ Carlo Fidani Regional Cancer Centre Trillium Health Partners Mississauga ON Canada

**Keywords:** automated prediction, hypofractionation, patient eligibility, prostate cancer

## Abstract

Clinical implementation of hypofractionated prostate radiotherapy (PROFIT trial, NCT003046759) represents an opportunity to significantly reduce the burden of treatment on the patient and clinic. However, efficacy was only demonstrated among the patient demographic who could meet the trial dose constraints and so it is necessary to emulate this triage step in clinical practice. The purpose of this study was to build a convenient tool to address the challenge of determining patient eligibility for hypofractionated treatment within the clinic. The tool was implemented within the Eclipse^TM^ treatment planning system using the scripting environment. Prior to planning a new case, the script computes and displays in a plot the fractional overlap of rectal and bladder wall with the planning target volume. Radial decision boundaries separate the plot into three zones and the new case is then classified as “feasible”, “uncertain”, or “not feasible”. The radial decision boundaries were derived from a retrospective analysis of the overlap values and dosimetric eligibility of 150 patients with intermediate risk prostate cancer. Two‐fold cross validation with repetitions demonstrated an average prediction accuracy of over 90%. The tool has been integrated into our clinical planning workflow to enable early identification of the need for planning consults and rapid a‐priori determination of dosimetric eligibility for hypofractionated radiotherapy. The tool can be readily adopted by other centres since the underlying metrics can be evaluated without scripting if desired.

## 1 INTRODUCTION

Dose‐escalated external beam radiotherapy (e.g., 7400–7800 cGy) using intensity modulated radiotherapy (RT) has been shown to improve biochemical control among patients with intermediate‐risk prostate cancer^1^ and so has become the standard of care. However, this approach involves prolonged treatment lasting 7–8 weeks and so increases the burden of treatment on both the patient and the clinic.

Therefore between 2006 and 2011 the multi‐institution PROFIT trial (NCT003046759) was conducted to compare a hypofractionated treatment course of 6000 cGy in 20 fractions to a conventional treatment of 7800 cGy in 39 fractions among intermediate‐risk patients. At a median follow‐up time of 5 yr, the hypofractionated treatment course was found to be non‐inferior to conventional treatment with respect to disease control and normal tissue toxicity among the 1206 patients enrolled in the study.^2^

Clinical implementation of PROFIT‐trial hypofractionation represents an opportunity to significantly reduce the burden of treatment on the patient and clinic. However, the PROFIT trial employed strict dose constraints for the rectum and bladder in order to safeguard against the potentially increased risk of normal tissue toxicity in the hypofractionated study arm. Patients were excluded from the trial if they could not meet these constraints. Consequently, non‐inferiority was only demonstrated among the patient demographic whose anatomy was well‐suited towards meeting constraints and so it is necessary to emulate this triage step in clinical practice.

The purpose of this study was to build an automated decision‐support tool to address the challenge of determining patient eligibility for hypofractionated treatment within the clinic. We propose an anatomy‐based metric to provide a‐priori prediction of whether constraints can be met for a given patient. The predictive efficacy of the metric is evaluated using 2‐fold cross validation. We integrate the tool into the Eclipse treatment planning system (TPS) via scripting to provide convenient decision‐support to the clinic.

## 2 METHODS

### 2.A Patient data and treatment planning

6000 cGy/20 fraction treatment plans were generated retrospectively for 150 patients who had previously been treated for intermediate risk prostate cancer with a conventional fractionation of 7800 cGy/39 fractions. All target and organ at risk (OAR) contouring was performed as per the PROFIT trial guidelines. OAR contours included the rectal wall, bladder wall and femoral heads. The clinical target volume (CTV) was defined as the prostate plus the proximal seminal vesicles if clinically indicated. The planning target volume (PTV) was equal to the CTV plus a 1 cm margin in all directions except posteriorly where a 7 mm margin was applied.

The Eclipse^TM^ TPS version 11 (Varian Medical Systems, Paolo Alto, CA, USA) was used to generate volumetric‐modulate arc therapy treatment plans for all patients. The PROFIT trial defined a set of preferred and a set of accepted dose constraints where treatment plans were expected to at least meet the accepted constraints (Table 1). Accordingly, patients were considered to be “dosimetrically eligible” if their plans could meet the accepted constraints. If these constraints could not be met for a given patient by two physicists, the patient was considered to be “dosimetrically ineligible” for hypofractionation.

**Table 1:** Treatment planning dose constraints employed by the PROFIT trial. The “accepted” set of constraints was used in the present study. D_*X*_ represents the dose received by *x*% of the listed OAR or target volumes

Constraints	Rectal wall		Bladder wall		Femur	CTV	PTV
	D_30_	D_50_	D_30_	D_50_	D_05_	D_99_	D_99_
Preferred (cGy)	4600	3700	4600	3700	4300	6000	5700
Accepted (cGy)	4710	3790	4710	3790	4400	6000	5700

### 2.B Overlap metric

Initial local experience with generating PROFIT trial hypofractionated treatment plans found that rectal and bladder wall D30 constraints were often unmet for challenging cases. In the presence of high PTV‐OAR overlap, target coverage or OAR dose must necessarily be compromised since OARs are to receive much less than the prescription dose. In particular, it might be expected that constraints cannot be met if the PTV overlaps more than 30% of the rectal or bladder wall since 30% of these OARs are required to receive less than 4710 cGy. The fractional overlap of PTV with the rectal and bladder wall may therefore serve as a strong anatomical indicator for the ability to meet PROFIT‐trial dose constraints.

Let $f_{RW}$ and $f_{BW}$ represent the fraction of the rectal and bladder wall that overlap with the PTV respectively. We propose that $f_{RW}$ and $f_{BW}$ can be added in quadrature to obtain a simple single‐valued overlap metric $\sqrt{{f_{RW}}^{2}+{f_{BW}}^{2}}$ which can be used to differentiate between eligible and ineligible patients. This metric was evaluated for each of the 150 patients and then used for prediction of dosimetric eligibility and assessment of planning difficulty.

### 2.C Evaluation of predictive efficacy

Prediction of dosimetric eligbility was performed using a simple threshold technique where a patient would be classified as dosimetrically ineligible if their overlap metric was greater than or equal to a single threshold value. This threshold must be determined by searching for the cut‐off value which maximizes prediction of eligibility among a training dataset.

In this study, two‐fold cross‐validation is employed to obtain this threshold and evaluate its predictive efficacy. Training and testing sets included equal proportions of dosimetrically eligible and ineligible patients. The two‐fold cross‐validation was repeated 1000 times with different randomly selected training and testing sets for each repetition. The average classification threshold, sensitivity, specificity and accuracy were then computed. We also performed three additional cross validations (with repetitions), one of which used only $f_{BW}$ for prediction, another used only $f_{RW}$ for prediction and the final cross validation investigated the use of the PTV volume for prediction.

### 2.D Prediction tool

The prediction tool was created using the Eclipse^TM^ scripting environment to enable access by dosimetrists, physicists and other clinicians within the TPS. When the script is executed, it computes the fractional overlap values $f_{BW}$ and $f_{RW}$ for the current patient. The rectal wall, bladder wall and PTV must therefore be contoured prior to script execution. The script displays a 2‐dimensional plot where the patient's fractional overlap values are indicated by a point with x and y coordinates equal to $f_{BW}$ and $f_{RW}$ respectively. Accordingly, the radial distance of this point from the origin is equal to the patient's overlap metric. The plot is then separated into three different sections “feasible”, “uncertain” and “not feasible” using different radial decision‐boundaries where the radii have been defined by overlap metric values observed among the 150 patients within this study.

## 3 RESULTS

**Figure 1:**
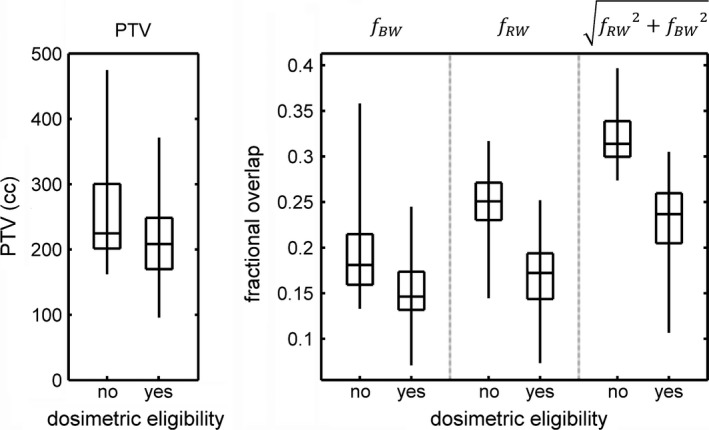
Distribution of PTV volume, bladder wall‐PTV fractional overlap (*f*_*BW*_), rectal wall‐PTV fractional overlap (*f*_*RW*_) and combined fractional overlap values for dosimetrically eligible and ineligible patients. The boxes span the 25th to 75th percentiles and the error bars indicate the range of each distribution.

6000 cGy/20 fraction treatment plans were generated for the 150 intermediate risk patients. 29.3% (*N* = 44/150) of patients were found to be dosimetrically ineligible (i.e., Table 1 constraints could not be met) due to high PTV‐bladder wall and PTV‐rectal wall overlap volumes. The distribution of PTV volumes, $f_{BW}$, $f_{RW}$, and overlap metric values for eligible and ineligible patients are summarized in Fig. 1. PTV volumes were found to be significantly different between eligible and ineligible patients using a Wilcoxon rank sum test (*P* < 0.01). All fractional overlap measures were also found to be significantly different between eligible and ineligible patients (*P* ≪ 0.001). The results from the two‐fold cross‐validation are summarized in Table 2.

**Table 2:** Average predictive efficacy and classification thresholds as evaluated using two‐fold cross‐validation with 1000 repetitions. Standard deviations are shown in brackets. True positives are defined as eligible patients correctly classified as eligible and true negatives are defined as ineligible patients correctly classified as ineligible

	PTV	*f*_*BW*_	*f*_*RW*_	$\sqrt{{f_{RW}}^{2}+{f_{BW}}^{2}}$
Sensitivity (%)	46.6	63.9 (5.3)	87.2 (5.2)	90.2 (2.0)
Specificity (%)	72.4	68.5 (7.4)	75.7 (3.8)	94.0 (3.5)
Accuracy (%)	54.2	65.2 (2.4)	83.8 (3.0)	91.3 (1.0)
Threshold	203.4 cc (11.9)	0.164 (0.006)	0.220 (0.008)	0.278 (0.004)

**Figure 2:**
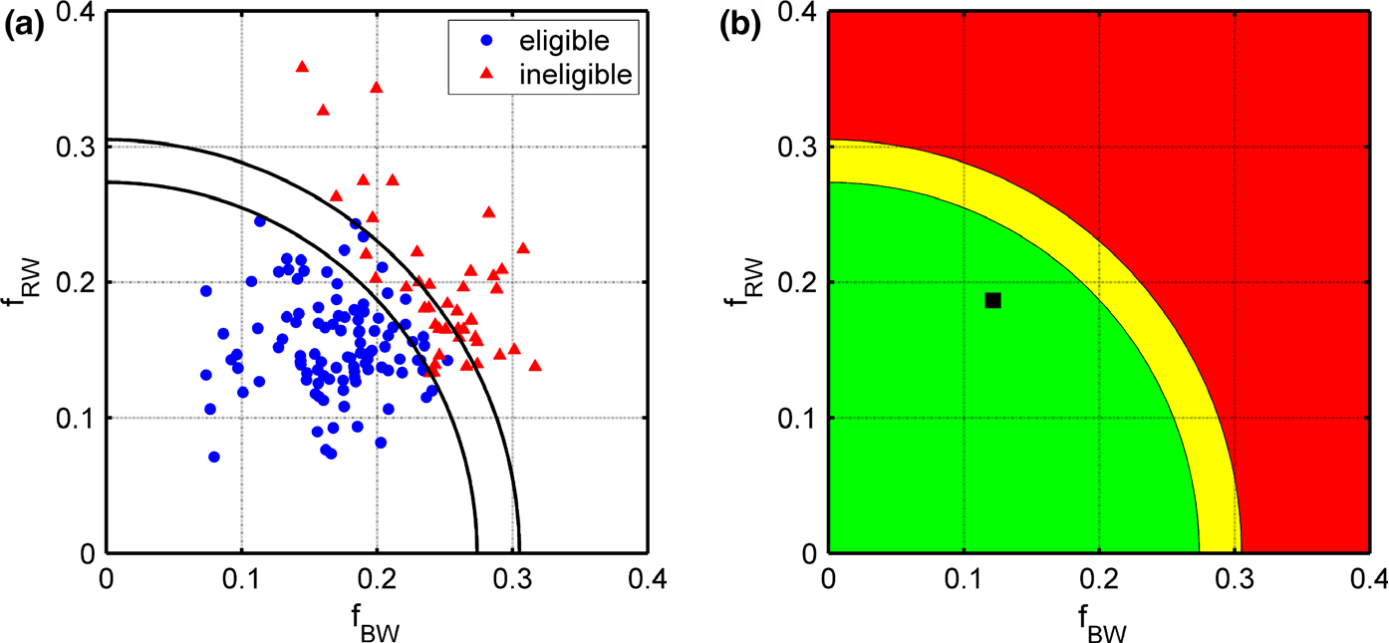
(a) Rectal wall‐PTV fractional overlap (*f*_*RW*_) versus bladder wall‐PTV fractional overlap (*f*_*BW*_) for dosimetrically eligible (*N* = 106/150) and ineligible (*N* = 44/150) patients. (b) Example of the decision support plot which appears within the TPS upon script execution. The patient's *f*_*BW*_ and *f*_*RW*_ values are indicated by a point (square marker) within the plot.

Figure 2 illustrates how the fractional overlap data from the *N* = 150 patients were used to build the decision support tool. Figure 2(a) shows a plot of $f_{RW}$ versus $f_{BW}$ for the *N* = 150 patients along with several radial boundaries. The first boundary is defined by a radius equal to the minimum overlap metric value observed among the ineligible patients (*r* = 0.274). The second boundary is defined by a radius equal to the maximum overlap metric value observed among eligible patients (*r* = 0.305).

Figure 2(b) provides an example of the plot that appears when the prediction tool is used to analyze a new patient within the TPS. The $f_{BW}$ and $f_{RW}$values for the new patient are indicated by a point in the plot (i.e., square marker). Dosimetric eligibility and planning difficulty for a new patient can then be evaluated at a glance by assessing the position of the point relative to the previously described radial boundaries.

If the point falls within the green zone as in Fig. 2(b), the new case is considered to be “feasible” since constraints could be met for 100% of patients in this study whose data points were located there (*N* = 95/150 in green zone). If the point fell within the yellow zone, the new case is referred to as “uncertain” since both eligible and ineligible patients were located in this zone (*N* = 25/150 in yellow zone). Only 11 of the 25 patients in the yellow zone were eligible and so meeting constraints for a new patient in this part of the plot would be expected to be very difficult. Finally, if the point fell within the red zone, the new case is referred to as “not feasible.” Meeting constraints is very unlikely for such a patient since 100% of patients located in this region were ineligible within this study (*N* = 30/150 in red zone).

## 4 DISCUSSION

The proposed tool summarizes the experience gained from generating 150 hypofractionated treatment plans and enables at‐a‐glance assessment of new patients relative to this prior knowledge. $f_{BW}$ and $f_{RW}$ values are indicated to assist in setting optimization priorities for difficult cases and maximally predictive assessment of dosimetric eligibility is possible by comparing a patient's overlap metric value to the threshold from Table 2. This threshold is very close to the radial boundary which defines the green zone (*r* = 0.278 versus 0.274) and so in practice the green zone boundary could be used for this purpose. Upon cross‐validation, the combined overlap metric that forms the basis of the tool was found to be the most robust predictor of dosimetric eligibility with a prediction accuracy exceeding 90% (Table 2).

This tool has now been integrated into our local clinical planning workflow. The script is run after contours are generated but prior to treatment planning. Significant effort is expended to meet constraints for any difficult cases located within the green zone (e.g., when a case is close to the green‐yellow boundary) since the prior knowledge from this study suggests that this is feasible. If a new case is located in the yellow zone, planning is still attempted but inter‐disciplinary discussions regarding planning feasibility are initiated in parallel since it may not be possible to meet constraints for these cases. Finally, hypofractionated treatment planning is not attempted for cases located in the red zone. The planner immediately notifies the radiation oncologist that hypofractionation is “not feasible” and the patient prescription is reverted to a conventional 78 Gy in 39 fraction regimen.

This work should enable triage of difficult cases to experienced planners, early identification of a need for planning consults and rapid determination of dosimetric eligibility for hypofractionated radiotherapy. 20% of patients would be classified as “not feasible” within the present study and so it is anticipated that unproductive treatment planning efforts will be avoided for 1 in 5 cases. The tool should also facilitate new planner education since training cases can be provided in order of increasing difficulty as indicated by the overlap metric.

The tool should be straightforward for other centres to adopt without scripting if desired since fractional overlap volumes can be computed manually within a TPS, added in quadrature with a calculator, and then compared to known decision thresholds. Users should be aware that the optimal classification thresholds for each centre are expected to exhibit some dependence on local factors such as planning expertise and contouring philosophy. However, we would expect these dependencies to be relatively minor with minimal deviation from a threshold value of 0.30 since an OAR‐PTV overlap of 30% precludes D30 constraints from being met while maintaining PTV coverage. Regardless, the overall approach of using a combined overlap metric to predict eligibility can be adopted where planners evaluate the overlap metric and accrue experience over time regarding whether constraints can be met at their centre for different cases. Users should also note that the definition of dosimetric eligibility in this study is only related to the PROFIT trial dose constraints and so the tool may not accurately predict ineligibility due to other factors such as small bowel dose or plan robustness.

The nearly 30% ineligibility rate reported in this study may or may not reflect the experience at other centres. The ineligibility rate is influenced by many factors such as radiation oncologist contouring philosophy and bowel and bladder preparation procedures. Local pre‐planning triage practices could also decrease the number of ineligible patients for whom planning is attempted in turn reducing the observed ineligibility rate at the planning stage (e.g., exclusion based on seminal vesicle involvement). The overall benefit of the tool reduces as the ineligibility rate decreases since there are fewer infeasible cases for which planning can be avoided. However, the combined overlap metric is still expected to be predictive within this context due to the direct relationship between OAR‐PTV fractional overlap and D30 constraints.

In the future, treatment planning may be fully automated for many sites.^3^ This would supersede the utility of the tool presented here since there would be no cost associated with generating treatment plans. However, in the medium term, many clinics may not have access to robust automated treatment planning and can therefore benefit from simple and effective treatment planning tools that require no specialized software or expertise.

## CONFLICT OF INTEREST

The authors declare that they have no conflicts of interest.
